# Author Correction: Microridge-like structures anchor motile cilia

**DOI:** 10.1038/s41467-024-52323-4

**Published:** 2024-09-23

**Authors:** Takayuki Yasunaga, Johannes Wiegel, Max D. Bergen, Martin Helmstädter, Daniel Epting, Andrea Paolini, Özgün Çiçek, Gerald Radziwill, Christina Engel, Thomas Brox, Olaf Ronneberger, Peter Walentek, Maximilian H. Ulbrich, Gerd Walz

**Affiliations:** 1 Department of Medicine IV, University Freiburg Medical Center, Faculty of Medicine, University of Freiburg, Hugstetter Strasse 55, 79106 Freiburg, Germany; 2 Faculty of Biology, University of Freiburg, Schaenzlestrasse 1, 79104 Freiburg, Germany; 3 Pattern Recognition and Image Processing, Department of Computer Science, University of Freiburg, Georges-Köhler-Allee 52, 79110 Freiburg, Germany; 4 BIOSS Centre for Biological Signalling Studies, University of Freiburg, Schänzlestrasse 18, 79104 Freiburg, Germany; 5 CIBSS Centre for Integrative Biological Signalling Studies, University of Freiburg, Schänzlestrasse 18, 79104 Freiburg, Germany

Correction to: *Nature Communications* 10.1038/s41467-022-29741-3, published online 19 April 2022

The original version of this Article inadvertently omitted references to previous work and the names of principal investigators who created Addgene plasmids described in ‘Buckley, C. E. et al. Reversible Optogenetic Control of Subcellular Protein Localization in a Live Vertebrate Embryo. Dev. Cell 36, 117–126 (2016)’ and ‘Yang, X., Jost, A. P.-T., Weiner, O. D. & Tang, C. A light-inducible organelle-targeting system for dynamically activating and inactivating signaling in budding yeast. Mol. Biol. Cell 24, 2419–2430 (2013)’. These have been added as references 66 and 67 at the ninth sentence of the ‘DNA reagents’ section of the Methods.

The ninth sentence of the ‘DNA reagents’ section of the Methods incorrectly read ‘Plasmids containing PIF6 and PHYB-mCherryCAAX (Plasmid #154913 and #51567, addgene, 490 Aresenal Way, Suite 100, Watertown, MA 02472) were subcloned into VF10 vectors with GFP, ezrin FERM domain (residues 1–310), or ezrin C-terminal domain (CTD) (residues 297–582) containing the phospho-mimetic T563E mutation.’

The correct ninth sentence of the ‘DNA reagents’ section of the Methods is: ‘Plasmids containing PIF6 and PHYB-mCherryCAAX (Plasmid #154913 created by Jonathan Clarke^66^ and #51567 created by Chao Tang^67^, addgene, 490 Aresenal Way, Suite 100, Watertown, MA 02472) were subcloned into VF10 vectors with GFP, ezrin FERM domain (residues 1–310), or ezrin C-terminal domain (CTD) (residues 297–582) containing the phospho-mimetic T563E mutation.’

These have been corrected in the PDF and HTML versions of the Article.

