# Supplementary material for: Microridge-like structures anchor motile cilia
Source: Nat Commun. 2022 Apr 19;13:2056. doi: 10.1038/s41467-022-29741-3 (PMC9018822; doi:10.1038/s41467-022-29741-3)
Supplement: Supplementary file 1 — Supplementary Information [file 41467_2022_29741_MOESM1_ESM.pdf]

# Microridge-like structures anchor motile cilia

Takayuki Yasunaga, Johannes Wiegel, Max D. Bergen, Martin Helmstädter, Daniel Epting, Andrea Paolini, Özgün Çiçek, Gerald Radziwill, Christina Engel, Thomas Brox, Olaf Ronneberger, Peter Walentek, Maximilian H. Ulbrich, Gerd Walz*

***corresponding author**

## SUPPLEMENTARY INFORMATION:

### SUPPLEMENTARY FIGURES 1-13 AND FIGURE LEGENDS

# SUPPLEMENTARY FIGURES AND FIGURE LEGENDS

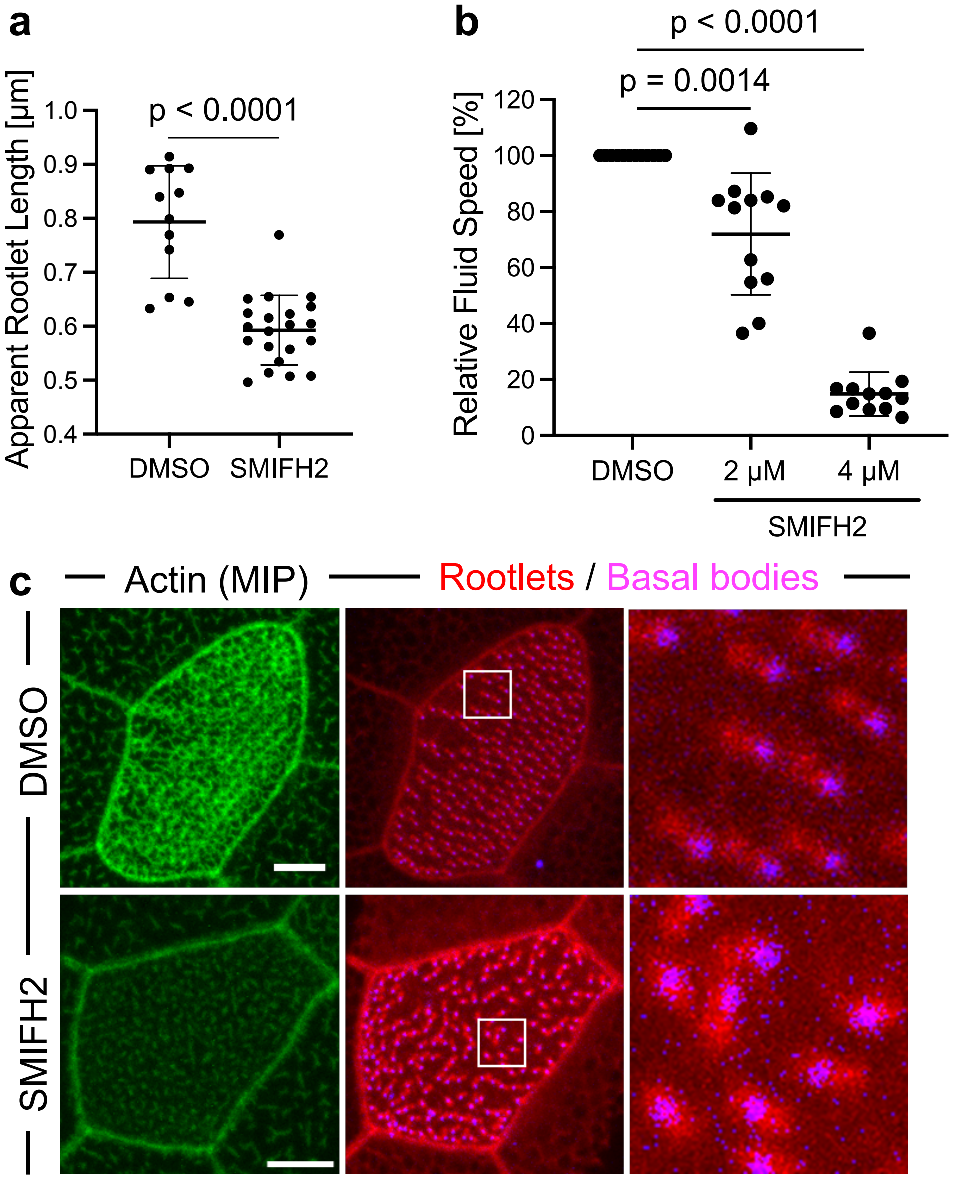


**Supplementary Figure 1 | The Formin inhibitor SMIFH2 prevents rootlet lengthening, fluid flow and polarization. a** The Formin inhibitor SMIFH2 (4 µM) prevents apparent rootlet lengthening (4 embryos, 21 cells) in comparison to DMSO control (2 embryos, 12 cells) (mean ± SD; Mann-Whitney-U test), and **b** reduces fluid flow generated by multiciliated cells in a dose-dependent manner (12 embryos each condition; mean ± SD; Mann-Whitney-U test). Source data are provided as a Source Data file. Representative data of at least three independent experiments. **c** SMIFH2 prevents polarization of basal bodies (BFP2-Centrin, magenta; Clamp-RFP, red; actin filaments, labeled by phalloidin, green) (scale bars, 5 µm).

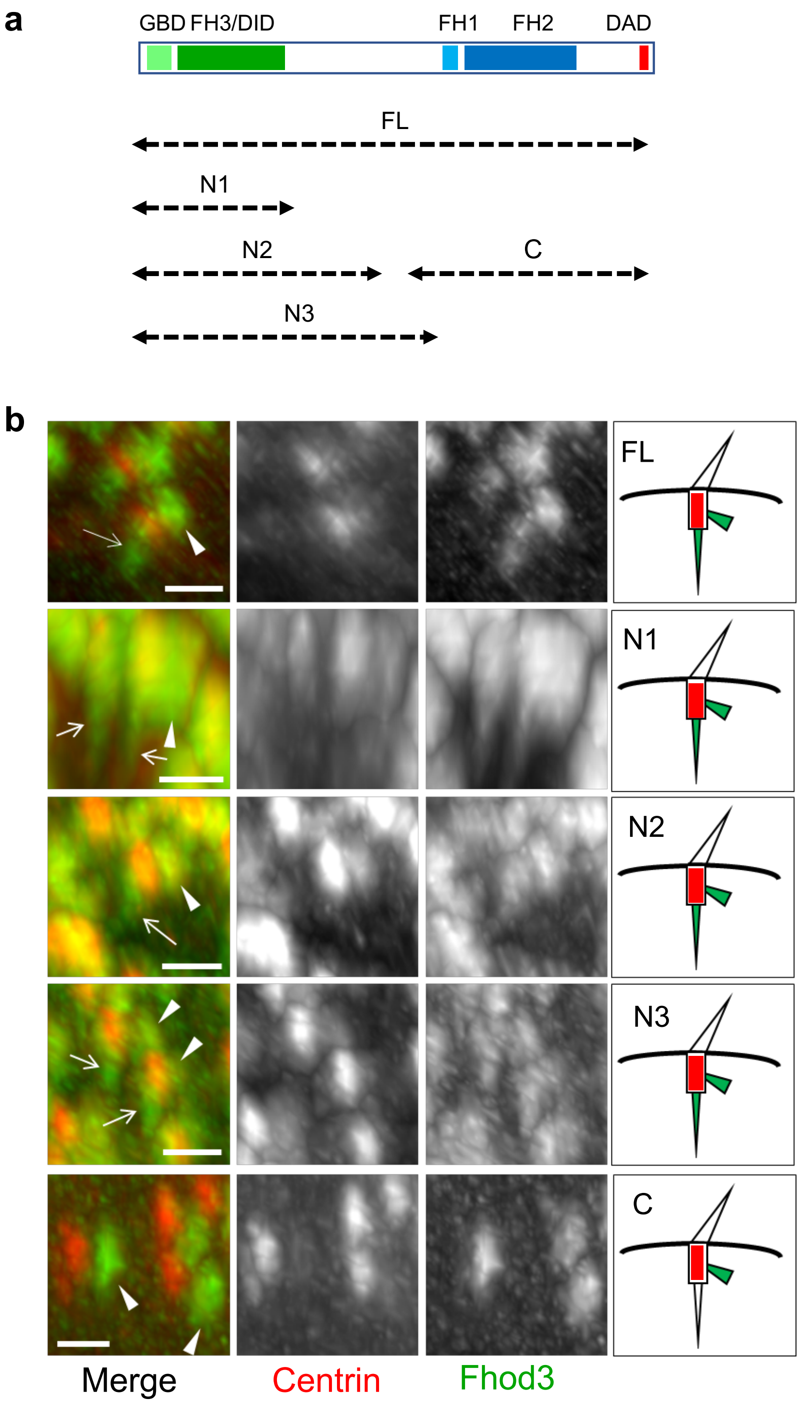


**Supplementary Figure 2 | Localization of *Xenopus* Fhod3 truncations in multiciliated cells.** **a** GFP-tagged Fhod3 truncations were generated by fusing GFP to the N-terminus. N1 corresponds to amino acid 1 to 504, N2 to amino acid 1 to 703, N3 to amino acid 1 to 942 and C to amino acid 768 to 1506 of *Xenopus* Fhod3. FL, full-length *Xenopus* GFP-Fhod3. **b** The N-terminal domain of Fhod3 is required for association with anterior and posterior rootlet structures in multiciliated cells, while the C-terminal domain localizes exclusively to the anterior rootlet. Images were reconstructed in 3D using IMARIS, and presented as depicted in the diagrams (scale bars, 1 µm).

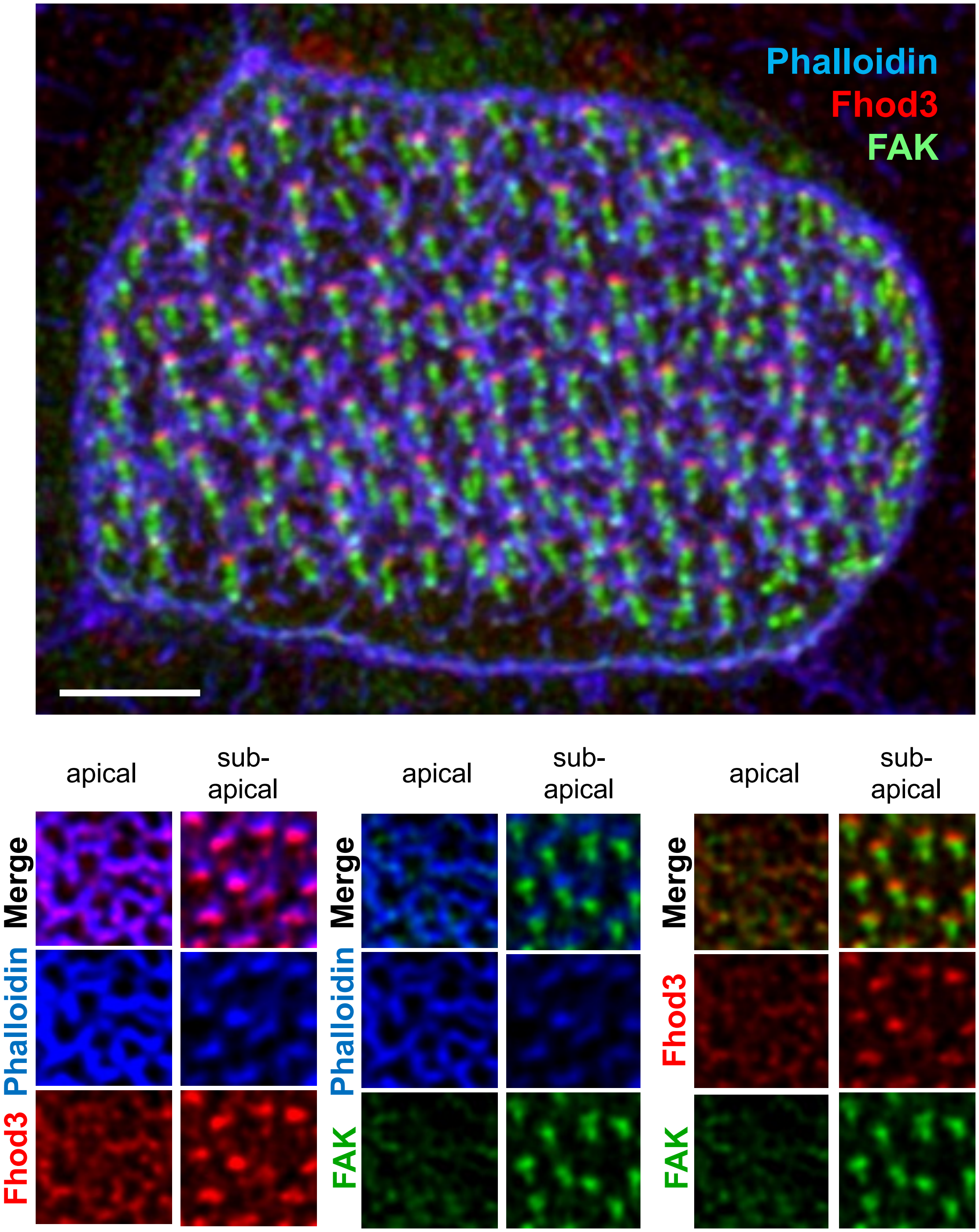


**Supplementary Figure 3 | Fhod3 localizes to the anterior rootlet within the subapical actin layer.** The upper panel is a maximal intensity projection with, depicting the localization of FAK (green), Fhod3 (red) and phalloidin (blue). The lower panels are single optical sections of the apical actin and subapical actin layer, depicting the position of Fhod3 in relationship to phalloidin (left panel), the position of FAK in relationship to phalloidin (middle panel), and the position of FAK in relationship to Fhod3 (right panel). Fhod3 partially overlaps with FAK at the distal tip of the anterior rootlet in the subapical actin layer (scale bar, 5 μm).

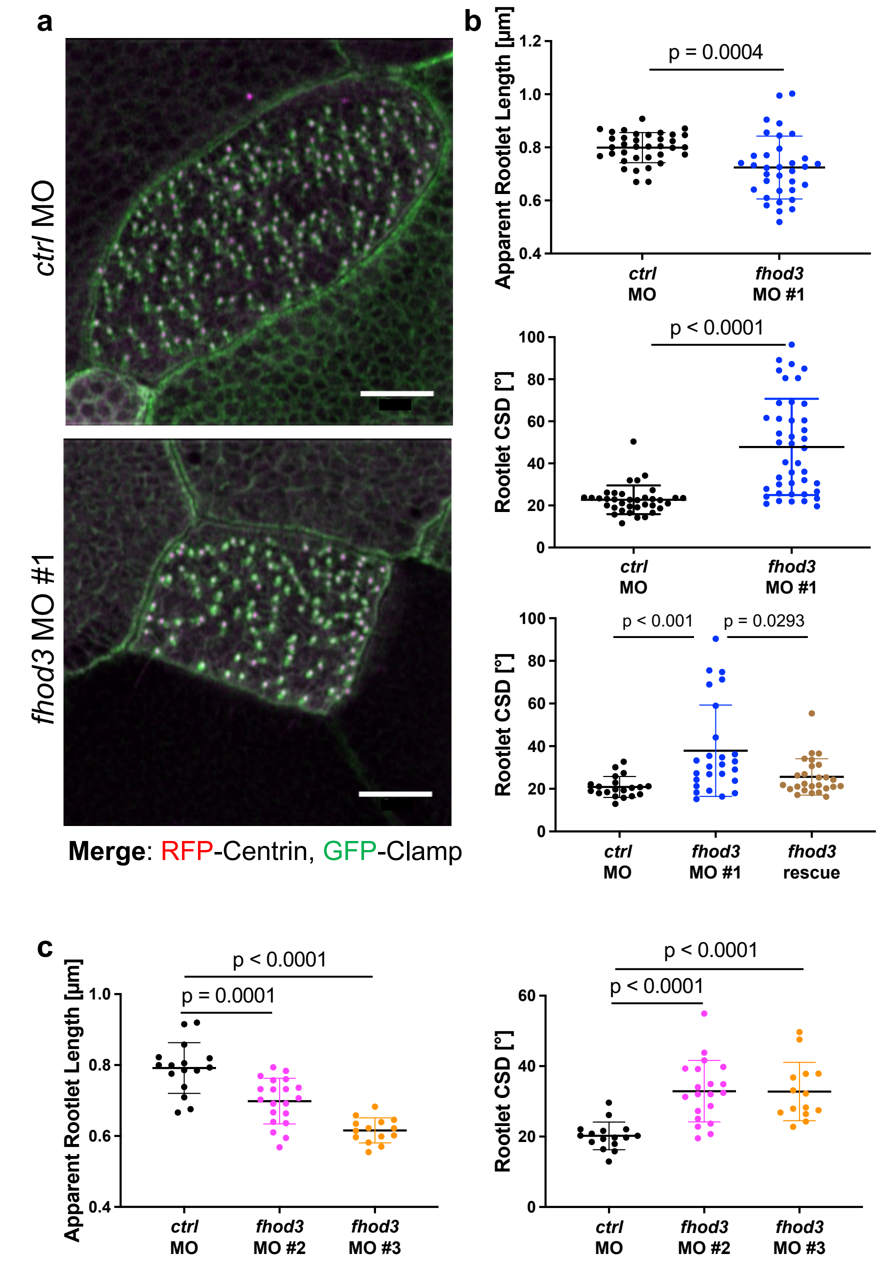


**Supplementary Figure 4 | Depletion of *Xenopus fhod3* affects rootlet length and rootlet polarity. a**. Depletion of *fhod3* with a splice -blocking morpholino oligonucleotide (MO) (*fhod3* MO #1) affected spacing and position of basal bodies. Control (*ctrl*) MO (11 embryos, 36 cells) and *fhod3* MO #1 (1 pmol) (12 embryos, 34 cells) were co-injected with 150 pg GFP-Clamp and 100 pg RFP-Centrin mRNA (scale bars, 5 µm). **b** Quantitative analysis revealed defective rootlet tilting, determined by an apparent decrease in rootlet length, and defective rootlet orientation, determined by an increase in rootlet circular standard deviation (CSD). To rescue the defective basal body orientation, 200 pg of *fhod3* full-length mRNA were co-injected with 1 pmol *ctrl* MO and *fhod3* MO #1 (*ctrl*: 7 embryos, 20 cells; *fhod3* MO#1: 8 embryos, 26 cells, *fhod3* rescue: 8 embryos, 26 cells). **c** The changes in rootlet length and orientation were replicated with two additional MOs (fhod3 MO #2 and fhod3 MO #3), using 3 pmol for each MO as well as for the *ctrl* MO. Between 2 and 5 *Xenopus* embryos were analyzed for each condition. The depicted symbols represent the results of a single multiciliated cells, encompassing the result of 100-200 measurements (mean ± SD; Mann-Whitney-U test). Source data are provided as a Source Data file.

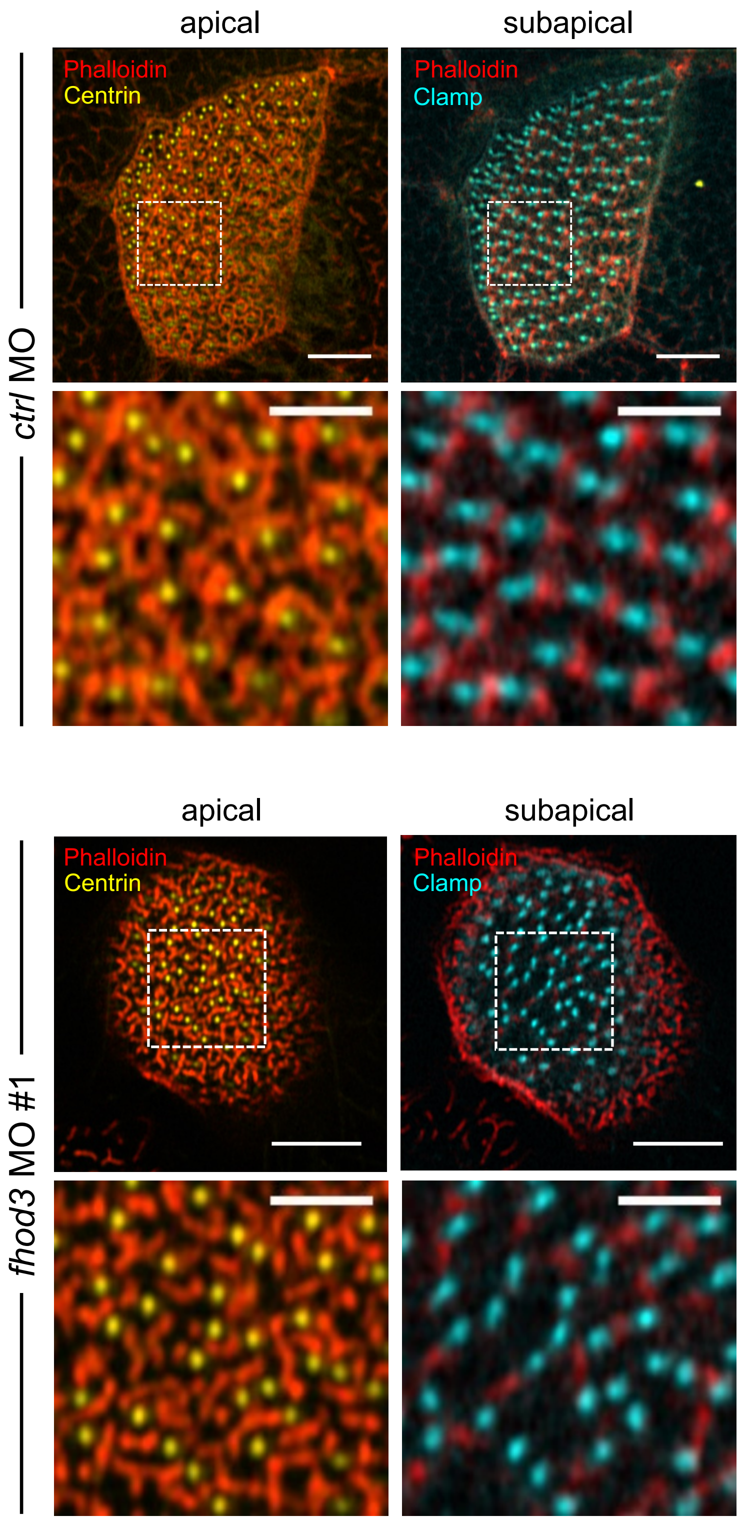


**Supplementary Figure 5 | Depletion of *Xenopus fhod3* primarily targets the subapical actin layer.** Depletion of *fhod3* with a splice -blocking morpholino oligonucleotide (MO) (*fhod3* MO #1) reduced the actin filaments primarily within the subapical actin layer, while the apical actin network was largely preserved. RPF-Centrin (Centrin, yellow) and GFP-Clamp (Clamp, turquoise) were used to label basal bodies and rootlets, respectively. Actin was labelled, using Phalloidin staining (scale bars upper panels, 5 µm; scale bars of the magnifications, 2 µm).

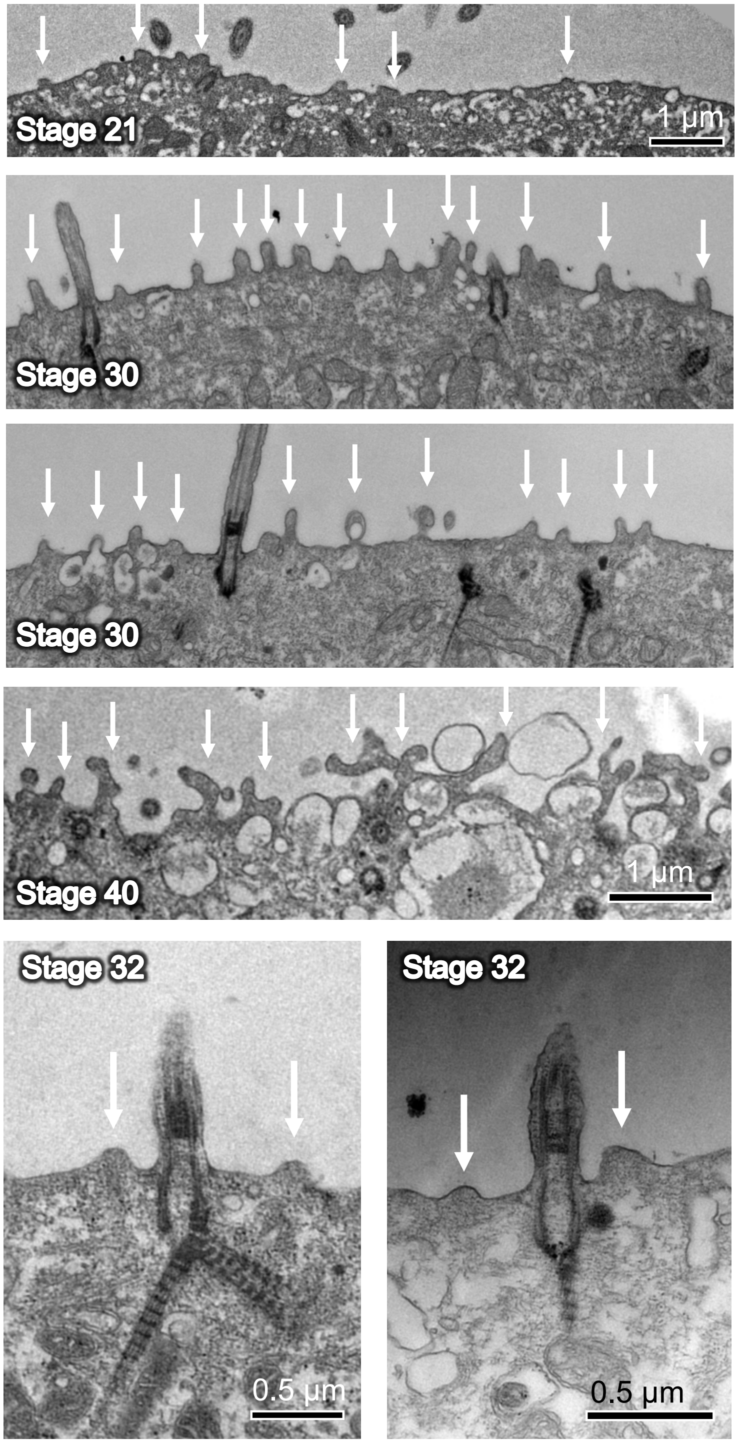


**Supplementary Figure 6 | Microridges on the apical cell surface of multiciliated cells**
Transmission electron microscopy (TEM) displays regularly spaced membrane protrusions (white arrows), starting to emerge at stage 21 (white arrows). At stage 30, microridges (white arrows) are fully developed. A tilted section is depicted for stage 40, highlight the elaborate membrane protrusions (white arrows), while cilia are disassembled. Membrane protrusions surrounding the ciliary axoneme at stage 32 (white arrows).

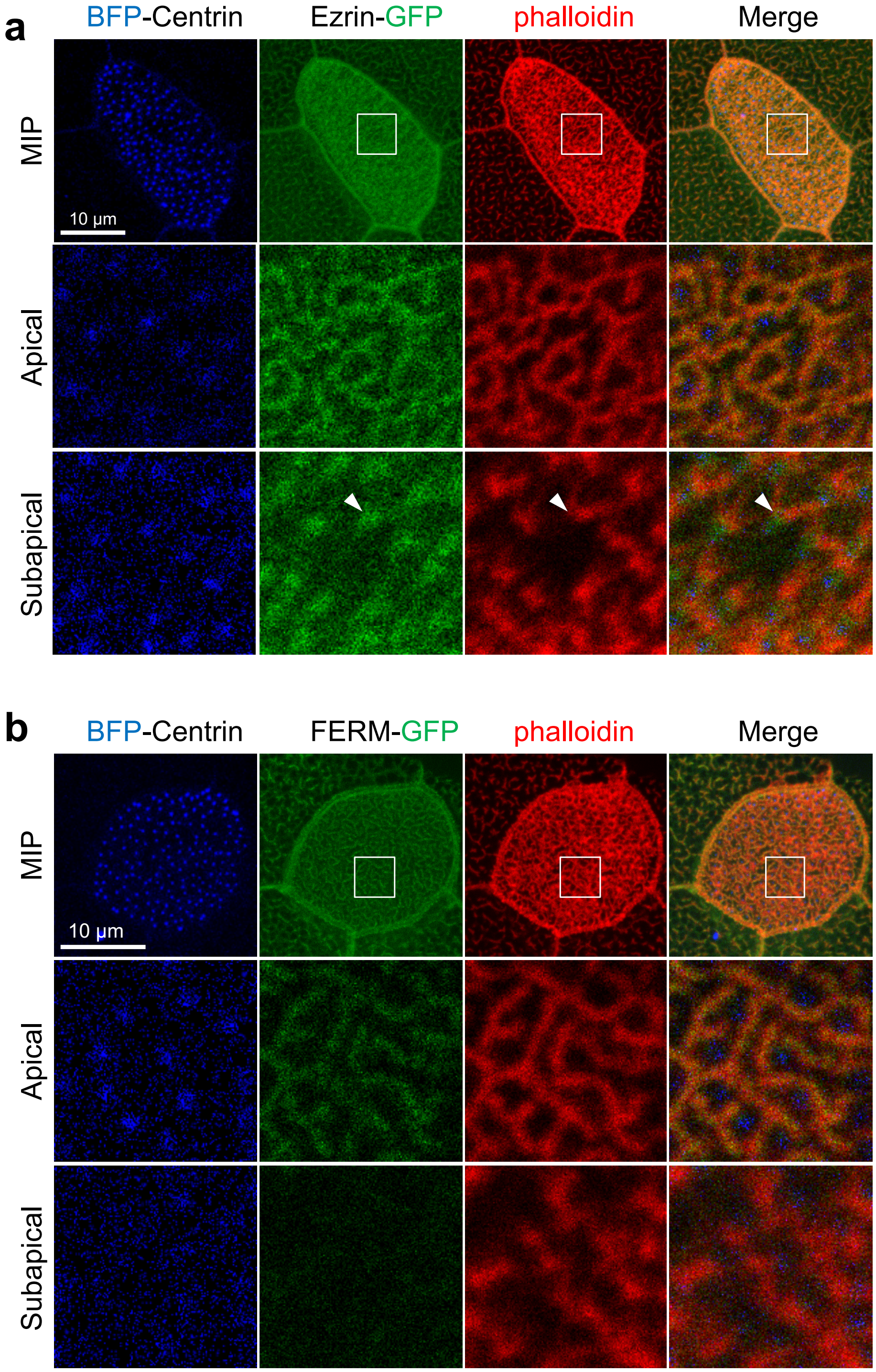


**Supplementary Figure 7 | Ezrin localizes to the actin cytoskeleton in multiciliated cells.** **a** Full-length Ezrin, fused to GFP (Ezrin-GFP), localized to the apical and subapical actin cytoskeleton. Basal bodies were labelled by BFP (blue), and the actin cytoskeleton by phalloidin (red). **b** The FERM domain of Ezrin, fused to GFP (FERM-GFP), localized almost exclusively to the apical actin layer. Basal bodies were labelled by BFP (blue), and the actin cytoskeleton by phalloidin (red).

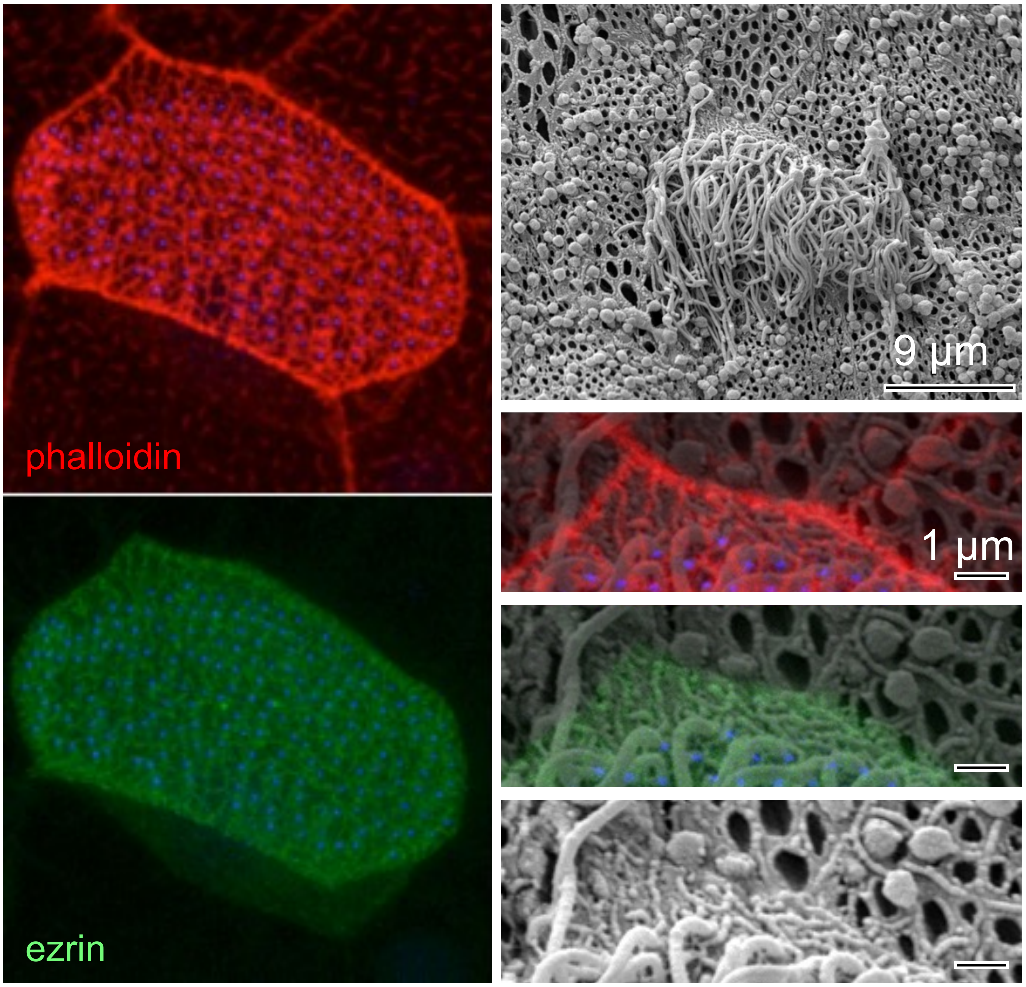


**Supplementary Figure 8 | Correlation between the actin cytoskeleton and microridges detected by scanning electron microscopy.** Correlative microscopy was performed by first generating phalloidin-stained immunofluorescent images. These images were subsequently analyzed by SEM, and overlayed.

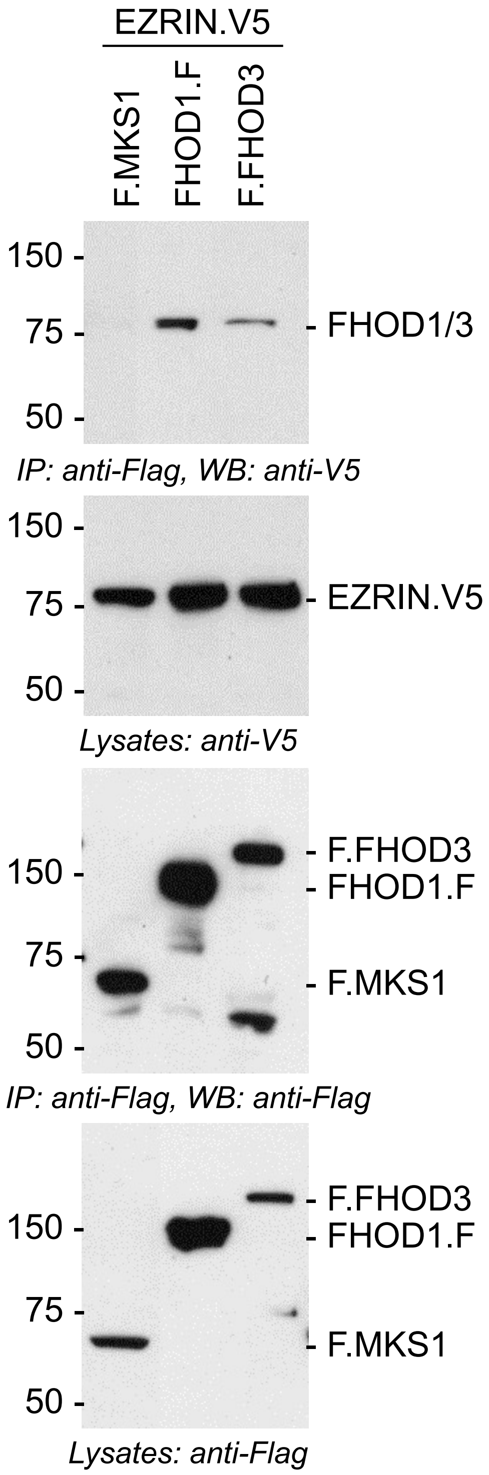


**Supplementary Figure 9 | EZRIN co-immunoprecipitates with FHOD1 and FHOD3.** Mammalian EZRIN, tagged with V5, was co-expressed with Flag-tagged FHOD1 or FHOD3 in HEK 293T cells. Flag-tagged MKS1 was used as a control. Flag-tagged proteins were precipitated, using M2 beads. Western blot analysis was performed with the antibodies as indicated.

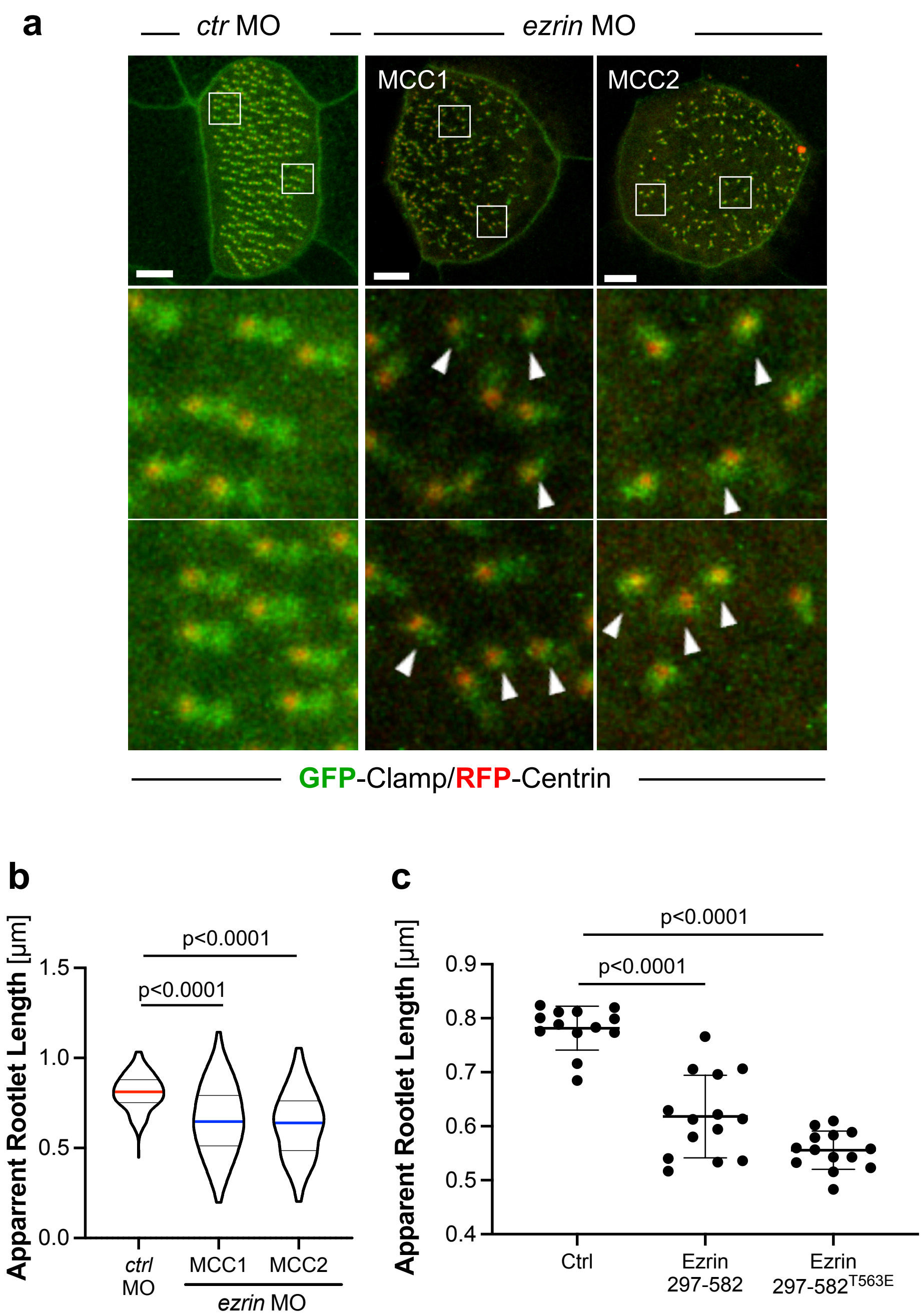


**Supplementary Figure 10 | Knockdown of Ezrin and overexpression of the actin-binding domain of Ezrin prevent rootlet anchoring. a** Depletion of *ezrin* results in shortening of the rootlet, marked by GFP-Clamp. The inserts demonstrate the shortening of rootlets (white arrows) of two multiciliated cells (MCC1 and MCC2) (scale bars, 5 µm). **b** Quantification revealed significantly reduced rootlet length in both MCCs, indicative of defective rootlet anchoring (Violin plot, showing the distribution of the apparent rootlet length with mean ± 1$^{st}$ and 3$^{rd}$ quartile; Mann-Whitney-U test). **c**, Expression of the C-terminal actin-binding domain of Ezrin$^{297\text{-}582}$ (3 embryos, 14 cells) and Ezrin$^{297\text{-}582(T563E)}$ (3 embryos, 14 cells), containing the phospho-mimetic T563E mutation, significantly reduced the length of the rootlet in comparison to γ-Tubulin-injected control (Ctrl) embryos (3 embryos, 13 cells), expressed at identical concentrations (600 ng) (Mann-Whitney-U test). Source data are provided as a Source Data file.

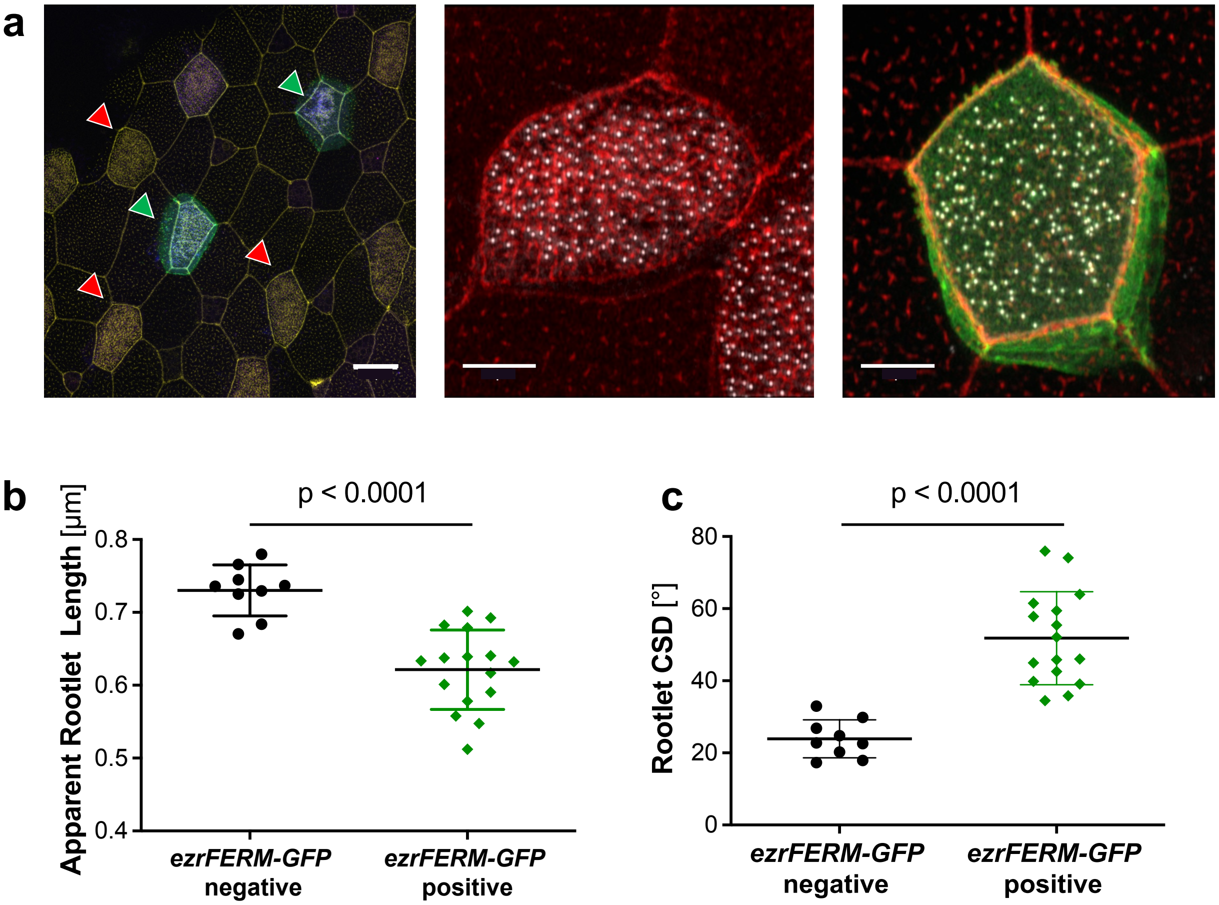


**Supplementary Figure 11 | The FERM domain of Ezrin exerts dominant-negative effects in a cell-autonomous manner. a** To generate a mosaic expression pattern, the N-terminal fragment of GFP-labeled *ezrin* (200 pg *MS2.αTub:ezrNT*-GFP) was co-injected with BFP-*clamp* (300 pg) and RFP-*centrin* (100 pg) mRNA. The red arrowheads in the left panel depict cells that lack expression of N-terminal ezrin fragment (an example is depicted in the middle panel), while the green arrowheads point towards *ezrNT*-GFP expressing cells (an example is depicted in the right panel) (scale bar left panel, 15 µm, scale bars middle and right panel 5 µm). **b** Apparent rootlet length was significantly decreased in *ezrNT*-GFP expressing cells. **c** Circular standard deviation (CSD) of rootlets was significantly increased in *ezrNT*-GFP expressing cells, indicated defective rootlet positioning (GFP-negative: 4 embryos, 9 cells; GFP-positive: 5 embryos, 16 cells) (mean ± SD; Mann-Whitney-U test). Source data are provided as a Source Data file.

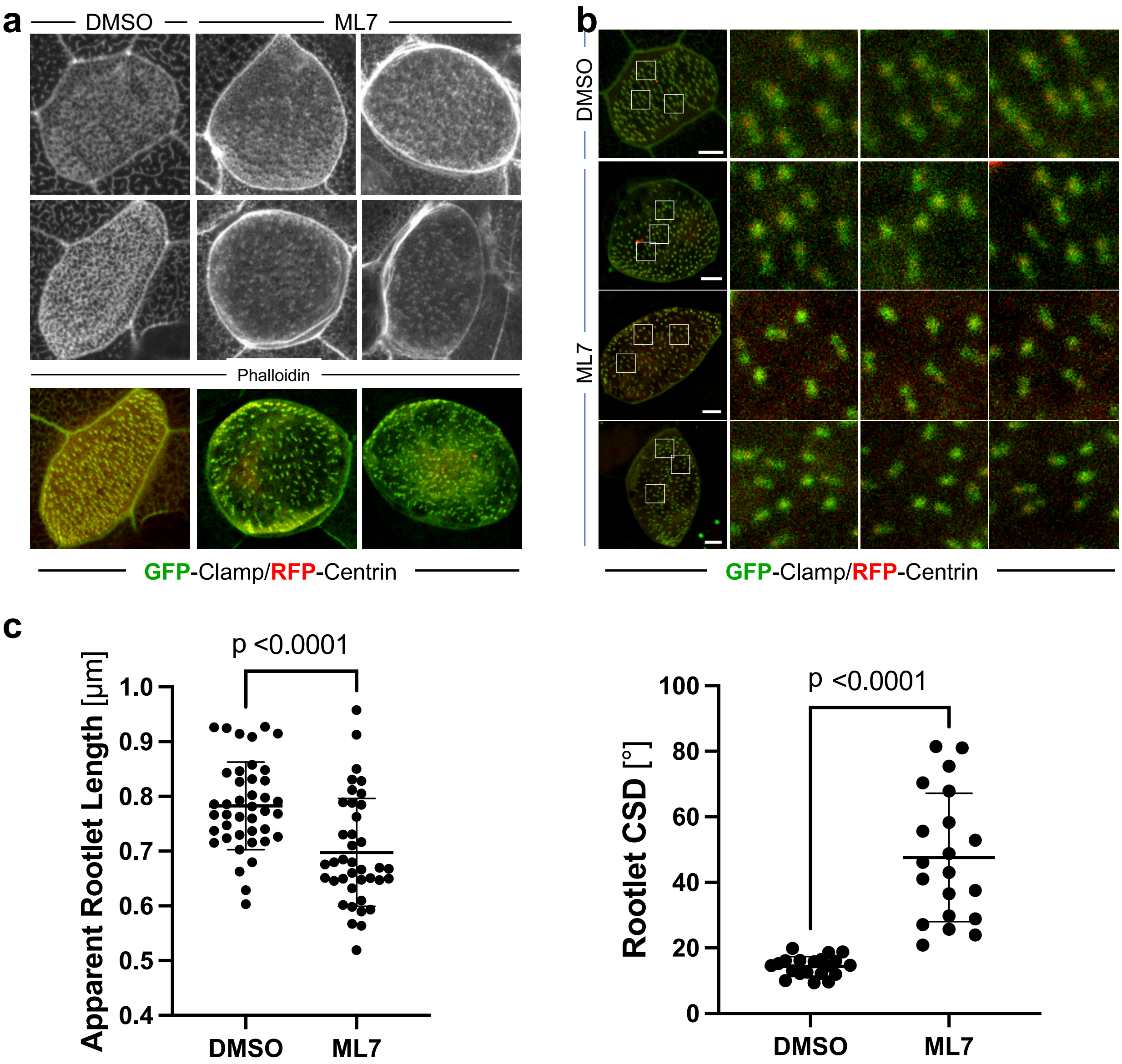


**Supplementary Figure 12 | Inhibition of myosin light chain kinase disrupts microridge formation. a** ML7 (40 µM) treatment, in comparison to the DMSO control, caused a reduction and an abnormal distribution of basal bodies (scale bars, 5 µm). **b** ML7 affected rootlet orientation (scale bars, 5 µm). **c**, Quantification revealed that ML7 decreased apparent rootlet length (DMSO: 8 embryos, 41 cells; ML7: 7 embryos, 39 cells) and increased rootlet circular standard deviation (CSD) (DMSO: 4 embryos, 20 cells; ML7: 4 embryos, 20 cells), consistent with impaired rootlet tilting and polarization (mean ± SD; Mann-Whitney-U test).

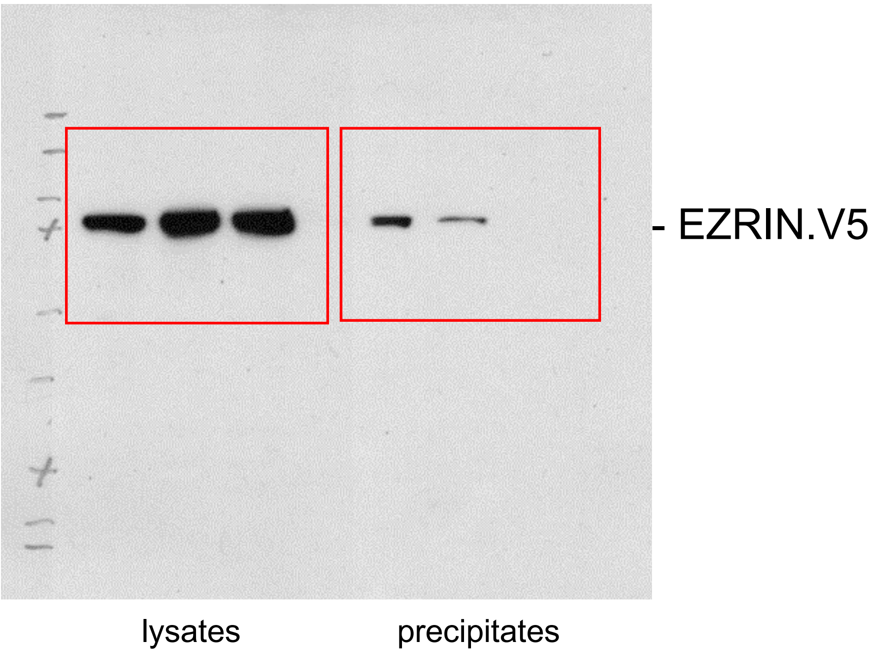


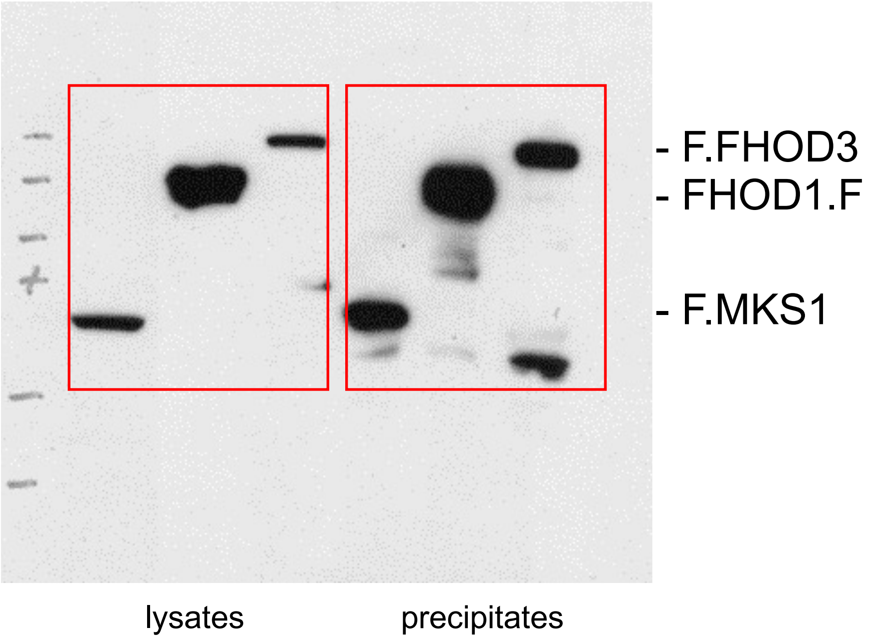


**Supplementary Figure 13 | Uncropped Western blots corresponding to Supplementary Fig. 9**
