# Supplementary material for: Microridge-like structures anchor motile cilia
Source: Nat Commun. 2022 Apr 19;13:2056. doi: 10.1038/s41467-022-29741-3 (PMC9018822; doi:10.1038/s41467-022-29741-3)
Supplement: Supplementary file 3 — Description of Additional Supplementary Files [file 41467_2022_29741_MOESM3_ESM.pdf]

DESCRIPTION OF ADDITIONAL SUPPLEMENTARY FILES

File Name: Supplementary Movie 1
3D reconstruction of cilia at stage 20-21 and stage 30, analyzed by transmission electron microscopy.

File Name: Supplementary Movie 2
Workflow, describing the analyses of cilia by transmission electron microscopy and 3 D reconstruction
