# Supplementary material for: Microridge-like structures anchor motile cilia
Source: Nat Commun. 2022 Apr 19;13:2056. doi: 10.1038/s41467-022-29741-3 (PMC9018822; doi:10.1038/s41467-022-29741-3)
Supplement: Supplementary file 6 — Reporting Summary [file 41467_2022_29741_MOESM6_ESM.pdf]

nature portfolio

Corresponding author(s): Gerd Walz

Last updated by author(s): Mar 18, 2022

Reporting Summary

Nature Portfolio wishes to improve the reproducibility of the work that we publish. This form provides structure for consistency and transparency in reporting. For further information on Nature Portfolio policies, see our Editorial Policies and the Editorial Policy Checklist.

Statistics

For all statistical analyses, confirm that the following items are present in the figure legend, table legend, main text, or Methods section.

n/a	Confirmed	
☐	☒	The exact sample size (*n*) for each experimental group/condition, given as a discrete number and unit of measurement
☐	☒	A statement on whether measurements were taken from distinct samples or whether the same sample was measured repeatedly
☐	☒	The statistical test(s) used AND whether they are one- or two-sided<br>*Only common tests should be described solely by name; describe more complex techniques in the Methods section.*
☒	☐	A description of all covariates tested
☐	☒	A description of any assumptions or corrections, such as tests of normality and adjustment for multiple comparisons
☐	☒	A full description of the statistical parameters including central tendency (e.g. means) or other basic estimates (e.g. regression coefficient) AND variation (e.g. standard deviation) or associated estimates of uncertainty (e.g. confidence intervals)
☐	☒	For null hypothesis testing, the test statistic (e.g. *F*, *t*, *r*) with confidence intervals, effect sizes, degrees of freedom and *P* value noted<br>*Give P values as exact values whenever suitable.*
☒	☐	For Bayesian analysis, information on the choice of priors and Markov chain Monte Carlo settings
☒	☐	For hierarchical and complex designs, identification of the appropriate level for tests and full reporting of outcomes
☒	☐	Estimates of effect sizes (e.g. Cohen's *d*, Pearson's *r*), indicating how they were calculated

Our web collection on statistics for biologists contains articles on many of the points above.

Software and code

Policy information about availability of computer code

Data collection	Zeiss Zen Black2.3; Leica LAS X 3.5.6.21594
Data analysis	ImageJ 1.53c/1.51s ; Graph Pad Prism 9 Version 9.1.0; RStudio Version 1.2.5033; Bitplane Imaris Version 9.6<br>2D U-Net MICCAI 2015: current versions are available at https://lmb.informatik.uni-freiburg.de/resources/opensource/unet/

For manuscripts utilizing custom algorithms or software that are central to the research but not yet described in published literature, software must be made available to editors and reviewers. We strongly encourage code deposition in a community repository (e.g. GitHub). See the Nature Portfolio guidelines for submitting code & software for further information.

Data

Policy information about availability of data

All manuscripts must include a data availability statement. This statement should provide the following information, where applicable:

- Accession codes, unique identifiers, or web links for publicly available datasets
- A description of any restrictions on data availability
- For clinical datasets or third party data, please ensure that the statement adheres to our policy

All data are available in the Source Data file.

Field-specific reporting

Please select the one below that is the best fit for your research. If you are not sure, read the appropriate sections before making your selection.

☒ Life sciences ☐ Behavioural & social sciences ☐ Ecological, evolutionary & environmental sciences

For a reference copy of the document with all sections, see nature.com/documents/nr-reporting-summary-flat.pdf

Life sciences study design

All studies must disclose on these points even when the disclosure is negative.

Sample size	Sample size was chosen based on comparable studies (e.g. Collins C, Kim SK, Ventrella R, Carruzzo HM, Wortman JC, Han H, Suva EE, Mitchell JW, Yu CC, Mitchell BJ. Tubulin acetylation promotes penetrative capacity of cells undergoing radial intercalation. Cell Rep. 2021;36 (7):109556; Lee M, Hwang YS, Yoon J, Sun J, Harned A, Nagashima K, Daar IO. Developmentally regulated GTP-binding protein 1 modulates ciliogenesis via an interaction with Dishevelled. J Cell Biol. 2019;218(8):2659-2676).
Data exclusions	No data were excluded.
Replication	The presented data represent the summary of several repitions as mentioned in Methods or Figure Legends.
Randomization	For quantitative analyses, Xenopus eggs were randomly asigned to either control or targeted morpholino-oligonucleotide injections
Blinding	Quantificaton was performed, using examiner-independent software.

Reporting for specific materials, systems and methods

We require information from authors about some types of materials, experimental systems and methods used in many studies. Here, indicate whether each material, system or method listed is relevant to your study. If you are not sure if a list item applies to your research, read the appropriate section before selecting a response.

Materials & experimental systems

n/a	Involved in the study	
☐	☒	Antibodies
☐	☒	Eukaryotic cell lines
☒	☐	Palaeontology and archaeology
☐	☒	Animals and other organisms
☒	☐	Human research participants
☒	☐	Clinical data
☒	☐	Dual use research of concern

Methods

n/a	Involved in the study	
☒	☐	ChIP-seq
☒	☐	Flow cytometry
☒	☐	MRI-based neuroimaging

Antibodies

Antibodies used	Anti-Flag M2 Affinity Gel (30 µl) (A2220), anti-Flag M2 antibody (F3165) (1:3000), anti-V5 (AB3792) (1:4000) from Sigma (Sigma-Aldrich Chemie GmbH, Eschenstraße 5, 82024 Taufkirchen, Germany)<br>Alexa Fluor 488 (A12379) –, Alexa Fluor 568 (A12380) –, Alexa Fluor 647 (A22287) -Phalloidin (1:5000) from Invitrogen (Invitrogen/ Thermo Fisher Scientific GmbH, Im Steingrund 4-6, 63303 Dreieich, Germany).
Validation	Anti-Flag: Anti-Flag: Brizzard, B.L., et al., BioTechniques, 16, 730 (1994); Knappik, A., and Pluckthun, A., BioTechniques, 17, 754 (1994); Chiang, C.M., and Roeder, R.G., Pept. Res., 6, 62 (1993); Current Protocols in Molecular Biology, Ausubel F.M., et al. (John Wiley and Sons Inc., NY, 1998), pp. 10.15.1.-10.16.29; Antibodies, A Laboratory Manual, Harlow, E. and Lane, D. (Cold Spring Harbor Laboratory Press, NY, 1988), pp. 514-517, 541-542, 547-549; Reichelt, P., et al., Protein Expression and Purification, 46, 483–488 (2006).<br>Anti-V5: e.g., Minoura I, Takazaki H, Ayukawa R, Saruta C, Hachikubo Y, Uchimura S, Hida T, Kamiguchi H, Shimogori T, Muto E. Reversal of axonal growth defects in an extraocular fibrosis model by engineering the kinesin-microtubule interface. Nat Commun. 2016 Jan 18;7:10058. doi: 10.1038/ncomms10058. PMID: 26775887; PMCID: PMC4735607.<br>Alexa-Phalloidin probes, see references in "The Molecular Probes Handbook" (https://www.thermofisher.com/de/de/home/references/molecular-probes-the-handbook/probes-for-cytoskeletal-proteins/probes-for-actin.html)

Eukaryotic cell lines

Policy information about cell lines

Cell line source(s)	HEK 293T/17 cells (ATCC CRL-11268)

Authentication	The cell line used was not authenticated.
Mycoplasma contamination	The cell line tested negative for mycoplasma contamination.
Commonly misidentified lines (See ICLAC register)	None

Animals and other organisms

Policy information about studies involving animals; ARRIVE guidelines recommended for reporting animal research

Laboratory animals	Xenopus laevis, obtained from EXRC, male and female, up to 5 ys.
Wild animals	This study did not involve wild animals.
Field-collected samples	This study did not involve field-collected samples.
Ethics oversight	All experiments were approved by the local authorities (Aktenzeichen 35-9185.81/G-17/62; Regierungspräsidium Freiburg, Germany).

Note that full information on the approval of the study protocol must also be provided in the manuscript.
